# Bovine serum albumin-templated nanoplatform for magnetic resonance imaging-guided chemodynamic therapy

**DOI:** 10.1186/s12951-019-0501-3

**Published:** 2019-05-20

**Authors:** Wei Tang, Hongbo Gao, Dalong Ni, QiFeng Wang, Bingxin Gu, Xinhong He, Weijun Peng

**Affiliations:** 1 Department of Radiology, Fudan University Shanghai Cancer Center, Shanghai, 200032 China; 2 Department of Oncology, Shanghai Medical College, Fudan University, Shanghai, 200032 China; 3 Departments of Radiology and Medical Physics, University of Wisconsin, Madison, WI 53705 USA; 4 Department of Pathology, Fudan University Shanghai Cancer Center, Shanghai, 200032 China; 5 Department of Nuclear Medicine, Fudan University Shanghai Cancer Center, Shanghai, 200032 China; 6 Shanghai Engineering Research Center of Molecular Imaging Probes, Shanghai, 200032 China; 7 Department of Interventional Radiology, Fudan University Shanghai Cancer Center, Shanghai, 200032 China

**Keywords:** Chemodynamic therapy, GSH, BSA-templated, MRI

## Abstract

**Background:**

Nanotechnology in medicine has greatly expanded the therapeutic strategy that may be explored for cancer treatment by exploiting the specific tumor microenvironment such as mild acidity, high glutathione (GSH) concentration and overproduced hydrogen peroxide (H_2_O_2_). Among them, tumor microenvironment responsive chemodynamic therapy (CDT) utilized the Fenton or Fenton-like reaction to produce excess hydroxyl radical (·OH) for the destruction of tumor cells. However, the produced ·OH is easily depleted by the excess GSH in tumors, which would undoubtedly impair the CDT’s efficiency. To overcome this obstacle and enhance the treatment efficiency, we design the nanoplatforms for magnetic resonance imaging (MRI)-guided CDT.

**Results:**

In this study, we applied the bovine serum albumin (BSA)-templated CuS:Gd nanoparticles (CuS:Gd NPs) for MRI-guided CDT. The Cu^2+^ in the CuS:Gd NPs could be reduced to Cu^+^ by GSH in tumors, which further reacted with H_2_O_2_ and triggered Fenton-like reaction to simultaneously generate abundant ·OH and deplete GSH for tumor enhanced CDT. Besides, the Gd^3+^ in CuS:Gd NPs endowed them with excellent MRI capability, which could be used to locate the tumor site and monitor the therapy process preliminarily.

**Conclusions:**

The designed nanoplatforms offer a major step forward in CDT for effective treatment of tumors guided by MRI.

**Electronic supplementary material:**

The online version of this article (10.1186/s12951-019-0501-3) contains supplementary material, which is available to authorized users.

## Introduction

The rapid development of nanomedicine has greatly accelerated the field of cancer theranostics due to the special physiochemical property of nanomaterials at the nanoscale. During the last decade, different kinds of nanomaterials have been cultivated for tumor diagnosis including computed tomography (CT) imaging [1], magnetic resonance imaging (MRI) [2, 3], single photon emission computed tomography (SPECT) imaging [4, 5], and positron emission computed tomography (PET) imaging [6, 7], and therapies such as photothermal therapy (PTT) [8–10], and photodynamic therapy (PDT) [11–13]. However, delicate tumor therapy should take full advantage of the specific tumor microenvironment (TME) including mild acidity, overproduced hydrogen peroxide (H_2_O_2_) and glutathione (GSH) [14]. Numerous nanomaterials with TME bioresponsive characteristics have emerged and flourished for cancer theranostics, such as acidity-sensitive drugs/gas molecules nanocarriers [15–17], acidity-sensitive function transformation nanoparticles [18–20], and hydrogen peroxide-sensitive nanoplatforms [21–23]. Among all the emerging strategies, chemodynamic therapy (CDT) is one of the most promising therapy methods that combines TME and traditional Fenton/Fenton-like reaction to realize a high logical and specific tumor treatment [24]. Briefly, catalytic ions trigger the disproportionation of H_2_O_2_ to generate high toxic ·OH to induce apoptosis of cancer cells. However, the concentration of H_2_O_2_ in TME is very limited for efficient CDT. Meanwhile, the high concentration of GSH [25] in TME could scavenge the produced ·OH, which strictly impede the efficiency of CDT. Therefore, it is worth to explore proper nanoplatforms to efficiently deplete the GSH and greatly enhance H_2_O_2_ concentration, leading to a double-enhanced CDT.

In addition, the nanoplatforms with imaging function is crucial for tumor diagnosis and treatment, which could be used for imaging-guided therapy or monitoring the corresponding therapeutic efficacy. Among the current imaging technology, magnetic resonance imaging (MRI) has a high spatial resolution, unlimited signal penetration depth, and exquisite soft tissue contrast. The nanoparticulate MRI contrast agents (CAs) have been widely exploited to improve the sensitivity of MRI. Therefore, developing nanoplatforms for MRI-guided CDT will undoubtedly assist the application of CDT and facilitate the potential clinical translation of this novel therapeutic strategy. With the above considered, the Gd and Cu-containing nanomaterials are promising candidates because Gd^3+^ could be used as CAs for *T*_1_-weighted MRI while Cu^2+^ ions could react with GSH to generate Cu^+^, triggering Fenton-like reaction for CDT.

**Fig. 1:**
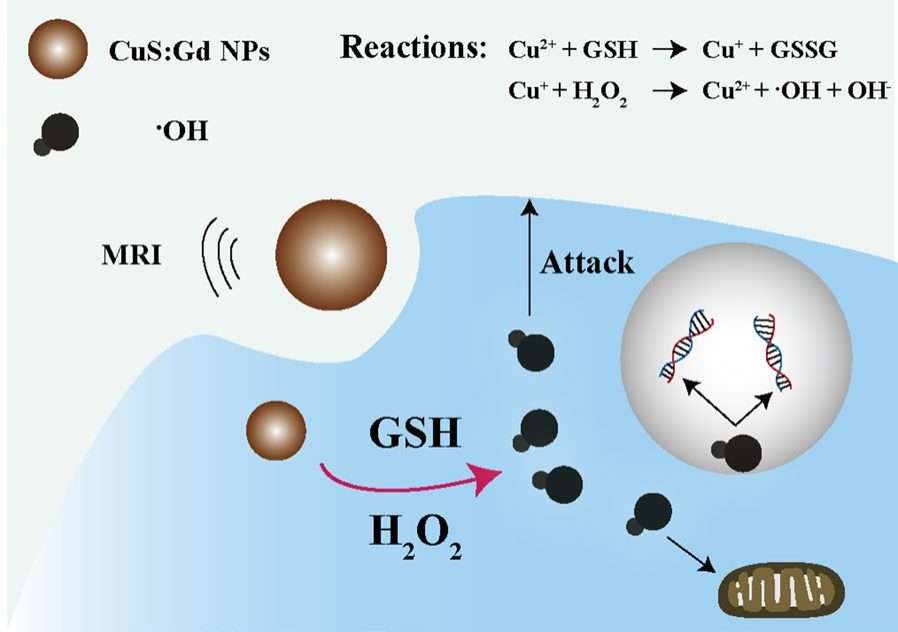
Schematic illustration of CuS:Gd NPs for MRI-guided CDT, the nanoparticles could simultaneously react with GSH and H_2_O_2_ to enhance the generation of ·OH for tumor CDT

Herein, the bovine serum albumin (BSA)-templated CuS:Gd nanoparticles (CuS:Gd NPs) were used for MRI-guided CDT, which could response tumor microenvironment selectively [26]. Different from other synthetic methods, this biomimetic synthesis method is widely used in the synthesis of nanomaterials which are used for bioapplications [27–29] due to the following advantages: (1) the synthesis procedures are very simple, (2) the synthesis process is very green and safe, (3) the synthesized nanoparticles are well dispersed with uniformity and excellent biocompability. As the Fig. 1 depicted, after endocytosis into the cancer cells, CuS:Gd NPs could react with GSH by reducing Cu^2+^ into Cu^+^, which reacts with H_2_O_2_ to generate ·OH to induce cancer cell death via Fenton-like reaction. Unlike traditional Fe-based nanoparticles for CDT [30, 31], the CuS:Gd NPs with GSH depletion could greatly enhance ·OH generation because the GSH would scavenge the produced ·OH to reduce CDT efficiency. Besides, the CuS:Gd NPs could act as MRI CAs to locate the tumor site and monitor the therapy process. The combination of MRI and CDT on the designed Cu/Gd-based nanoplatform not only enriches the library of CDT nano-agents but also provides enlightenment for further exploration of new-type CDT therapies.

## Experimental section

### Materials

Copper(II) chloride dihydrate (CuCl_2_·2H_2_O), gadolinium(III) chloride hexahydrate (GdCl_3_·6H_2_O), and sodium sulfide nonahydrate (Na_2_S·9H_2_O) were from Sigma-Aldrich. Albumin from BSA was bought from Sigma-Aldrich. Sodium hydroxide was obtained from Titans Chemical Reagent Co., Ltd. (Shanghai, China). All chemicals were used without any purification. Deionized water (18.2 MΩ cm resistivity at 298 K) was used through all the experiments.

## Synthesis BSA-templated CuS:Gd NPs

Firstly, 5 mL CuCl_2_·2H_2_O solution (0.01 mmol/mL), 1 mL GdCl_3_·6H_2_O solution (0.025 mmol/mL), and 5 mL BSA solution (50 mg/mL) were added in a one-neck flask (25 mL) with strong stirring in a oil bath (310 K). Then, 500 μL NaOH solution (1 M) was used to adjust the pH value to ~ 12 and the color of the mixture change into a deep blue. Later, 400 μL Na_2_S·9H_2_O (242.16 mg/mL) was quickly injected into the reaction system and the color turned into a deep brown. After 4 h reaction, the solution was dialyzed (MWCO = 8000–14,000 Da) against deionized water for 24 h to remove redundant Cu^2+^ and Gd^3+^. Besides, the powder was collected after lyophilization and redissolved in 3 mL PBS (1×, pH 7.4) for further experiments. The concentrations of Gd^3+^ and Cu^2+^ were calculated using ICP-OES (Agilent 725, Agilent Technologies).

### Characterization of CuS:Gd NPs

The transmission electron microscope (TEM) images of the CuS:Gd NPs were gained using JEM-2100. The confocal laser scanning microscopy (CLSM) images were recorded from the A1R microscope (Nikon Co.).

### The generation of ·OH with CuS:Gd NPs

The CuS:Gd NPs (20 mL, 50 mM) was mixed with 20 mL GSH solution (50 mM) and the intermediate precipitated product (described as CuS:Gd-G) was collected for the ·OH generation measurement. To measure the generation of ·OH, 10 μg/mL methylene blue (MB), 10 mM H_2_O_2_ were mixed at different pH (7.4, 6.5 and 5.4). After different time incubation, the ·OH generation ability was evaluated by MB degradation via the change in absorbance. Besides, electron paramagnetic resonance (EPR) spectrometer was used to ensure the existence of ·OH at corresponding conditions.

### Cell culture and cell viability evaluation

4T1 mouse breast tumor cell line and LO2 human normal liver cell line were cultured using the high-glucose DMEM (Gibco, USA), which was supplemented with 10% heat-inactivated fetal bovine serum (FBS) and 1% penicillin. The cell line was obtained from Shanghai Institute of cells, Chinese Academy of Sciences, and cultured at 310 K in a humid atmosphere with 5% carbon dioxide. The cell viabilities were monitored by a 3-[4,5-dimethylthiazol-2-yl-]-2,5-diphenyltetrazolium bromide (MTT) test and calcein/PI staining test following the manufacturer’s instructions.

### In vitro ROS generation detection

After co-culturing with 100 μg/mL of CuS:Gd NPs for 12 h, the cellular-ROS stress level was monitored by the ROS Assay Kit (Beyotime Biotechnology). Firstly, the cells were washed with PBS twice. Then, the cells were incubated with 2′,7′-dichlorodihydrofluorescein diacetate (DCFH-DA) (10 μM) for 30 min and washed for 3 times with PBS. Finally, the in vitro ROS generation ability of the CuS:Gd NPs was detected by the confocal fluorescence microscopy.

### Animal experiments

For the in vivo toxicity study, healthy 7-week old female Kunming mice (∼ 25 g) were bought and raised in Fudan University. Firstly, female Kunming mice were set into three groups (six mice for each group) randomly. After the i.v. injection of CuS:Gd NPs (dosage of 25 mg/kg in 100 µL), the mice were sacrificed in 3 days and 30 days respectively, and major organs (heart, liver, spleen, lung and kidney), blood were dissected for further pathological evaluation and biochemistry evaluation.

For the in vivo anti-tumor performance evaluation, 1 × 10^8^ 4T1 cells were injected into the Balb/c mice. When the tumor volume reached 100 mm^3^, the mice were randomly divided into 2 groups (n = 6) for further experiment. The tumor-bearing mice were intratumoral injected with (1) saline, (2) 5 mg/kg CuS:Gd NPs. In addition, the administration was repeated every 3 days. Every 3 days, the body weight and the tumor volume were recorded. The tumor volume was ensured according to the equation of V = L × W^2^/2 (L, the longest dimension; W, the shortest dimension). About 18 days later, all the mice were sacrificed, the tumor tissues and main organs were dissected for further H&E staining evaluation. For the in vivo MRI performance detection, the tumor bearing mouse was intravenously injected with 25 mg/kg CuS:Gd NPs and the MRI signals at different time points (0.5 h, 1 h, 3 h, 6 h and 12 h) were further monitored.

### Statistical analysis

The results were presented as the mean ± standard deviation. The statistical significance of the differences was determined by one-way ANOVA. Values with P < 0.05 were recognized as statistically significant (* means P < 0.05, ** means P < 0.01, *** means P < 0.001).

## Results and discussion

### Synthesis and characterization of CuS:Gd NPs

**Fig. 2:**
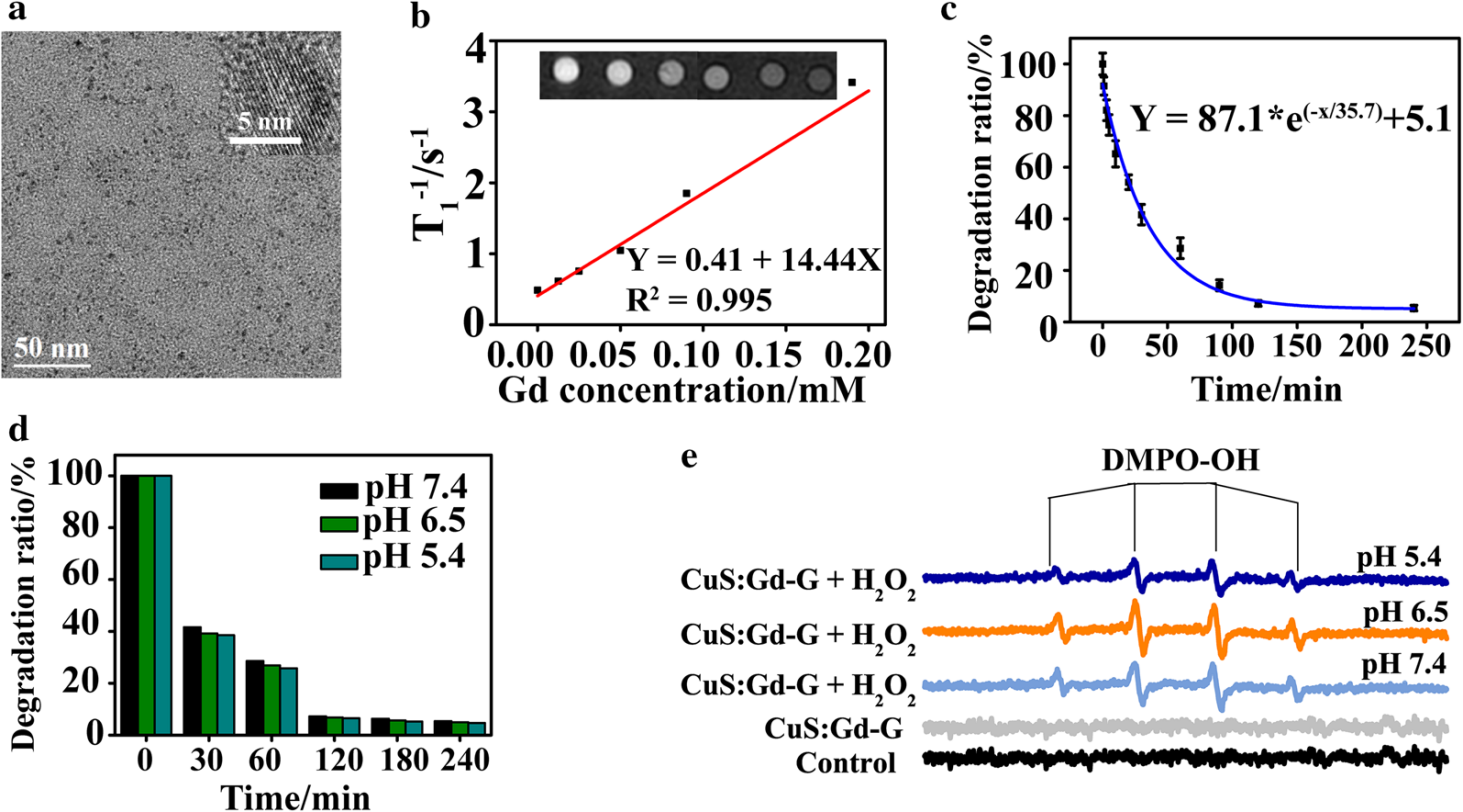
Characterization of CuS:Gd NPs. **a** TEM images of CuS:Gd NPs. **b** The longitudinal relaxation rate of the CuS:Gd NPs, Inset: Corresponding MR images. **c** The degradation performance of CuS:Gd NPs on MB at different time points (1 min, 3 min, 5 min, 10 min, 20 min, 30 min, 60, min, 90 min, 120 min and 240 min) (n = 3, mean ± SD). **d** The degradation of MB under different pH values (5.4, 6.5 and 7.4), indicating the negligible difference under different pH conditions. **e** EPR spectra of different groups (CuS:Gd-G only, CuS:Gd-G with H_2_O_2_ at pH 7.4, CuS:Gd-G with H_2_O_2_ at pH 6.5 and CuS:Gd-G with H_2_O_2_ at pH 5.4) with the 5,5-dimethyl-l-pyrroline *N*-oxide (DMPO) as the spin trap to detect the ·OH generation during the reaction

The CuS:Gd NPs were synthesized according to the previous literature. As shown from the TEM images, the synthesized CuS:Gd NPs was about 5 nm in diameter with well shape uniformity (Fig. 2a). To further understand the compositions, we collected the X-ray photoelectron spectroscopy (XPS) spectrum of the nanoparticles. As Additional file 1: Figure S1 depicted, the CuS:Gd NPs indeed had the elements of Cu, S and Gd. For the Cu element, the binding energy of 933.8 eV and 942.5 eV were attributed to the 2p_3/2_ of CuO and 935.2 eV were attributed to the 2p_3/2_ of CuS. Besides, the binding energy of 953.2 eV and 954.5 eV were the peaks of 2p_1/s_ of CuS [32]. The peaks of 163.7 eV and 164.8 eV represented the energy of 2p_3/2_ of CuS. Meanwhile, the peaks of 167.9 eV and 169.2 eV represented the energy of 2p_3/2_ of CuSO_4_, which showed partial oxidation. For the Gd element, the binding energy of 142 eV, 143.68 eV, 145.2 eV and 147.8 eV, 149.2 eV were attributed to the 4d_5/2_ and 4d_3/2_ of Gd_2_O_3_. Importantly, the CuS:Gd NPs presented well *T*_*1*_-weighted MRI performance and the longitudinal relaxivity (*r*_*1*_) was calculated to be 14.44 mM^−1^ s^−1^, which was about 4 times higher than that of the commercial Magnevist (Gd-DTPA, *r*_*1*_ = 3.217 mM^−1^ s^−1^) (Fig. 2b). The ·OH generation ability was then assessed using the traditional methylene blue (MB) degradation method via the variation of absorbance intensity at 664 nm. With the reaction time (1 min, 3 min, 5 min, 10 min, 20 min, 30 min, 60, min, 90 min, 120 min and 240 min) increased, the degradation ratio was increased and the MB was almost entirely degraded at 240 min after the reaction (Fig. 2c). Besides, the MB degradation experiments were also conducted at different pH (7.4, 6.5 and 5.4), the results showed little difference under different pH conditions (Fig. 2d), showing the weak pH-dependent tendency of CuS:Gd-G NPs.

Furthermore, we also used the electron paramagnetic resonance (EPR) spectrometer which utilized 5,5-dimethyl-l-pyrroline *N*-oxide (DMPO) as the spin trap agent to confirm the presence of ·OH. Under different pH values (pH 5.4, 6.5 and 7.4), the group of CuS:Gd-G NPs (100 µg/mL) containing H_2_O_2_ (100 µM) generated ·OH while the group of CuS:Gd-G NPs only did not produce ·OH (Fig. 2e). With all the results supported, the CuS:Gd NPs were certainly used for MRI-guided CDT, realizing the two functions of both GSH depletion and ·OH generation for a further anti-tumor application.

### In vitro cytotoxicity of CuS:Gd NPs and ROS generation performance

**Fig. 3:**
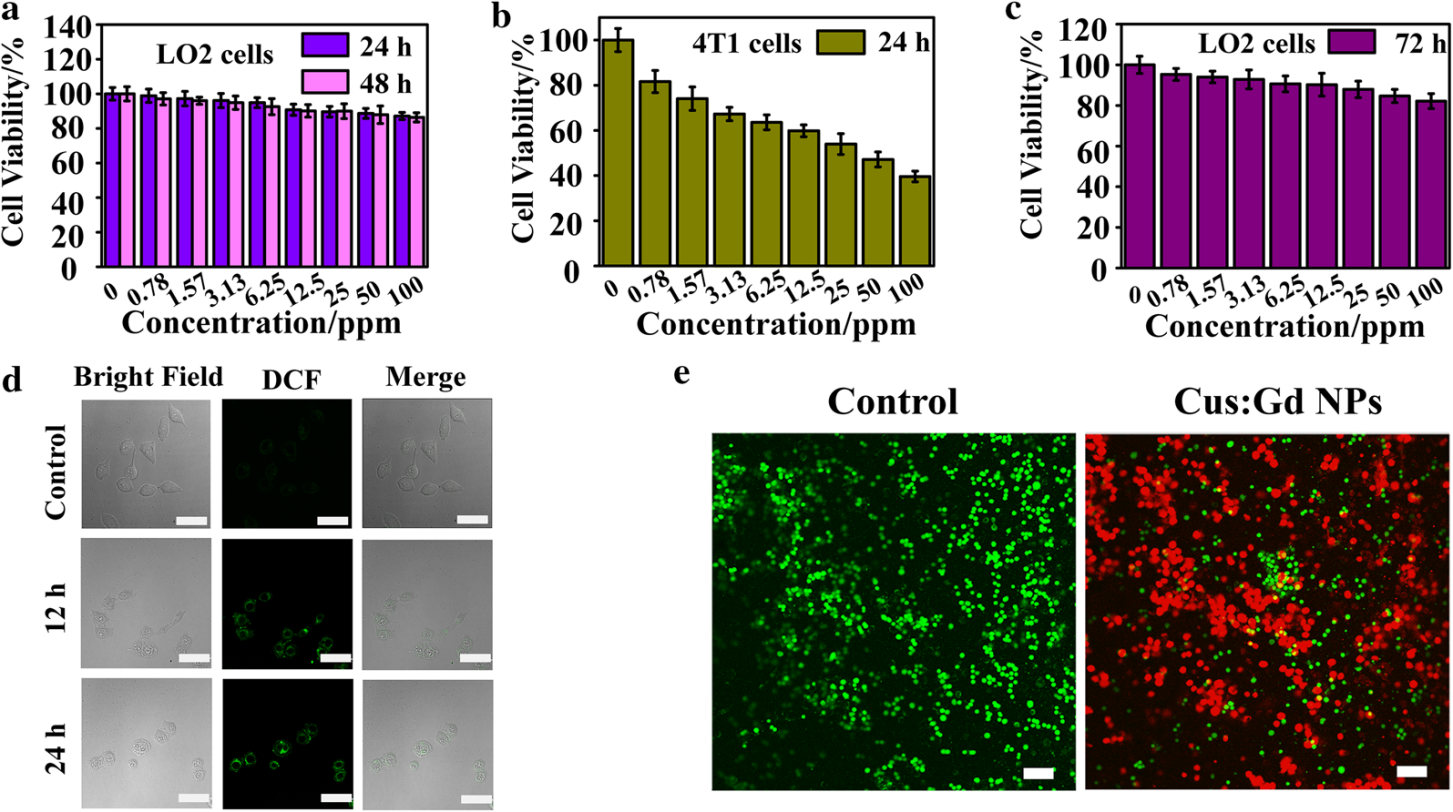
In vitro toxicity on normal cells and CDT performance on cancer cells of CuS:Gd NPs. **a** Cell viability of CuS:Gd NPs with different concentrations on the normal cells (LO2 cell) at 24 h and 48 h after co-incubation, demonstrating the well biocompatibility of CuS:Gd NPs. (n= 6, mean ± SD). **b** Cell viability of CuS:Gd NPs with different concentrations on the cancer cells (4T1 cell) at 24 h and 48 h after co-incubation (n= 6, mean ± SD). **c** Cell viability of CuS:Gd NPs with different concentrations on the normal cells (LO2 cells) at 72 h after co-incubation. (n= 6, mean ± SD). **d** Confocal images of DCFH-DA (2′,7′-dichlorofluorescein diacetate) stained 4T1 cells of two groups (control group and the CuS:Gd NPs group) at different times points. Scale bar: 50 μm. **e** Confocal images of the live and dead cells stained with calcein/PI of different groups (saline group and the CuS:Gd NPs group co-cultured with 4T1 cells for 24 h), demonstrating the well CDT capability of CuS:Gd NPs. Scale bar: 100 μm

The cell viability of normal and cancer cell lines that co-cultured with CuS:Gd NPs were measured via traditional MTT assay. As Fig. 3a and Additional file 1: Figure S2 depicted, the CuS:Gd NPs showed limited toxicity towards normal cells (LO2 cells, BRL cells and NRK cells) after 24 h and 48 h co-incubation, while they presented well anti-cancer cells performance under different pH values (Fig. 3b) after 24 h co-incubation. Moreover, cytotoxicity of CuS:Gd NPs on normal cells after long time co-incubation (72 h) were also negligible, which supported the further in vivo application of CuS:Gd NPs (Fig. 3c). The difference in toxicities between normal cells and cancer cells were caused by the difference of GSH amount in cells. In cancer cells, the CuS:Gd NPs would react with the high concentration of GSH and induced the Fenton-like reaction to generate ·OH, inducing cancer cells death. Subsequently, the confocal images showed that CuS:Gd NPs were able to be phagocytosed into cells after 6 h co-incubation and the ratio was increased after 12 h co-incubation (Additional file 1: Figure S3). To monitor the reactive oxygen species (ROS) generation in cellular level, two groups (control and CuS:Gd NPs) were set and the DCFH-DA probe was used. After 12 h co-incubation, the control group presented little fluorescence compared with the CuS:Gd NPs group (Fig. 3d) and the fluorescence intensity increased after 24 h co-incubation for the CuS:Gd NPs group, which is in consistent with the results from MB degradation and MTT results. Since the CuS:Gd NPs would generate ROS in cancer cells to induce cancer cells death, the calcein/PI probe was used to differentiate the live and dead cells after co-incubation. Compared with the control group, Cus:Gd NPs treated group exhibited great CDT efficiency on a cellular level (Fig. 3e).

### In vivo cytotoxicity of CuS:Gd NPs

Before the in vivo application of CuS:Gd NPs for cancer MRI-guided CDT, the evaluation of potential in vivo toxicity was necessary. The mice were randomly divided into three groups including the control group, the CuS:Gd NPs (25 mg/kg) injected group for short-term toxicity assessment (lasting 3 days) and the CuS:Gd NPs (25 mg/kg) injected group for long-term toxicity assessment (lasting 30 days). The mice weights were recorded every 3 days and the main organs of all groups were dissected after corresponding days for hematoxylin–eosin staining (H&E) staining. Meanwhile, we also focused on the blood biochemistry test including alkaline phosphatase (ALP), aminotransferase (AST), amino- transferase (ALT), and blood urea nitrogen (BUN) and the standard blood parameters, including red blood cells (RBCs), creatinine (CREA) indicators, red blood cell distribution width-standard deviation (RDW-SD), white blood cell (WBCs), hemoglobin (HGB), lymphocyte (LYM), hematocrit (HCT), mean corpuscular volume (MCV), mean corpuscular hemoglobin (MCH), and mean corpuscular hemoglobin concentration (MCHC). As shown in Additional file 1: Figures S4, S5 and S6, no obvious toxicities in vivo was found for mice injected with the CuS:Gd NPs, indicating well biosafety of this novel nanoplatforms.

### In vivo MRI performance and the in vivo CDT efficiency of CuS:Gd NPs

**Fig. 4:**
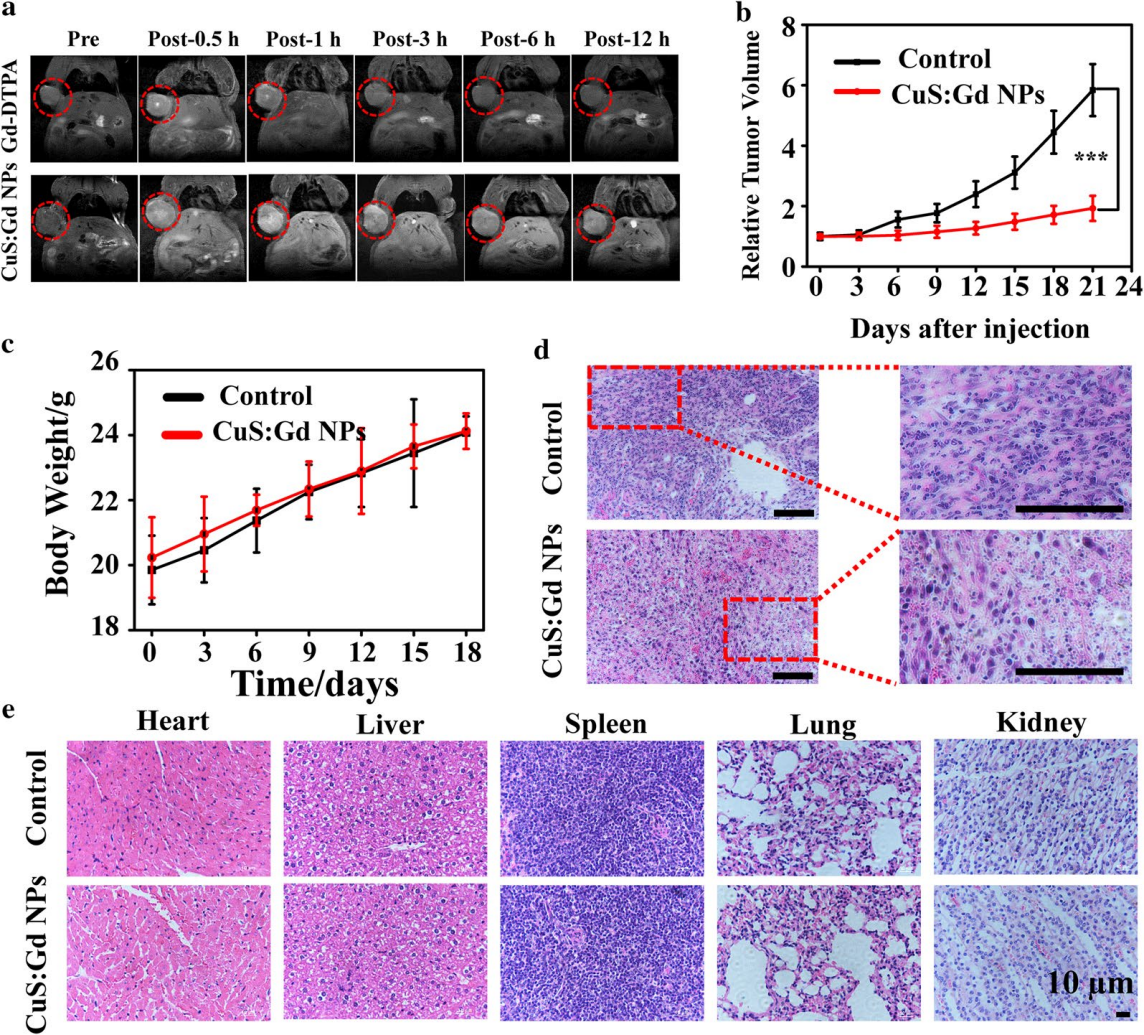
In vivo MRI performance and CDT ability of CuS:Gd NPs. **a** *T*_*1*_-weighted MR images of mice at different time points after the injection of CuS:Gd NPs (25 mg/kg) and the commercial Magnevist (Gd-DTPA) for comparison, confirming the well MRI behavior of CuS:Gd NPs in vivo. **b** Time course change of the tumors volume of different groups (saline group and CuS:Gd NPs group), indicating the excellent CDT performance of CuS:Gd NPs in vivo. (n= 6, mean ± SD). **c** Body weights change of the 4T1 tumor-bearing Balb/c mice during CDT process. (n= 6, mean ± SD). **d** H&E staining of the tumor tissues from the 4T1 tumor-bearing Balb/c mice from different groups. Scale bar: 50 μm. **e** H&E staining of the main organs after the treatment of CuS:Gd NPs

Based on the excellent *T*_*1*_-weighted MRI performance of CuS:Gd NPs in solution level, we evaluated the capability of CuS:Gd NPs as MRI CAs in vivo. The tumor-bearing mice were firstly intravenously injected with CuS:Gd NPs (25 mg/kg in 100 µL) or the commercial Magnevist, and the MR images were recorded after different post-injection time including 0.5 h, 1 h, 3 h, 6 h and 12 h. After the injection of CuS:Gd NPs, the MR enhancement at the tumor site kept increasing during 60 min (Fig. 4a), which was much stronger than that of Magnevist group. The significant increase of MR signal after injection was owing to the enhanced permeability and retention (EPR) effect during the blood circulation and the high *r*_*1*_ values of CuS:Gd NPs. Moreover, the MR signal remained very high even at 12 h after the injection while the MR signal of the Magnevist group fade, revealing the well MRI capability of CuS:Gd NPs in vivo to monitor the CDT efficiency.

With the good biosafety of CuS:Gd NPs considered, we then assessed the in vivo tumor CDT efficiency. The experiments were conducted on the tumor-bearing Balb/c mice. When the tumor volume reached 100 mm^3^, the two different groups were intratumorally injected with the corresponding saline (control group) or CuS:Gd NPs (5 mg/kg) every 3 days. After 21 days, the tumor volume significantly increased for the control group (with saline injected), while the tumor size was greatly decreased for the CuS:Gd NPs injected group (Fig. 4b). Meanwhile, the body weights of the two groups had no obvious difference during the treatment, indicating the biosafety of CuS:Gd NPs at the suitable dosage (Fig. 4c). Specifically, the tumor size of the control group increased from 100 to 600 mm^3^ and the tumor size of the CuS:Gd NPs injected group was significantly inhibited from 100 mm^3^ to less than 200 mm^3^, supporting the great CDT efficiency of CuS:Gd NPs. Moreover, the H&E staining of the tumor tissues of the two groups showed the apparent anti-tumor cells ability of CuS:Gd NPs. As Fig. 4d depicted, numerous cells in the CuS:Gd NPs-injected group were in the stage of necrosis or apoptosis, which confirmed that the CuS:Gd NPs would efficiently induce cancer cells death for well CDT performance in vivo. Moreover, the injections of CuS:Gd NPs with several times did not cause the potential toxicities for the main organs including heart, liver, spleen, lung and kidney and the adjacent normal tissues of tumor judging from the H&E staining images (Fig. 4e and Additional file 1: Figure S7).

## Conclusions

In summary, we synthesized and applied the BSA-templated CuS:Gd NPs for tumor MRI-guided CDT. Firstly, the CuS:Gd NPs owns the good performance on MRI to locate the tumor site and monitor the CDT process. Meanwhile, the nanoplatform could respond TME selectively, simultaneously consuming GSH and triggering the Fenton-like reaction for specific CDT of tumors. The generation of ·OH by CuS:Gd nanoplatforms could be greatly enhanced because of the GSH depletion. The in vivo results confirmed the great capabilities of the CuS:Gd NPs on tumor MRI and selectively therapy. As one of the Cu-based nanoplatforms, the CuS:Gd NPs not only enriched the CDT agents but also amplified the CDT effects on tumors with high specificity. In addition to the application of tumor imaging and therapy, the CuS:Gd NPs could also be utilized for antibacterial research as the bacterial microenvironment is similar to the tumor microenvironment. Besides, the nanoplatform could also act as the biosensor for monitoring the GSH concentration as the CuS:Gd NPs would react with GSH, causing the changes of Cu^2+^/Cu^+^ ratio and absorption spectrum.

## Additional file

**Additional file 1.** Additional figures.

## Data Availability

All data generated or analysed during this study are included in this published article and its additional files.

## Abbreviations

GSH: glutathione
CDT: chemodynamic therapy
BSA: bovine serum albumin
MRI: magnetic resonance imaging
NPs: nanoparticles
H_2_O_2_: hydrogen peroxide
TME: tumor microenvironment
