# Supplementary material for: Bovine serum albumin-templated nanoplatform for magnetic resonance imaging-guided chemodynamic therapy
Source: J Nanobiotechnology. 2019 May 20;17:68. doi: 10.1186/s12951-019-0501-3 (PMC6528315; doi:10.1186/s12951-019-0501-3)
Supplement: Supplementary file 1 — Additional file 1. Additional figures. [file 12951_2019_501_MOESM1_ESM.doc]

**Bovine Serum Albumin-templated Nanoplatform for Magnetic Resonance Imaging-Guided Chemodynamic Therapy**

Wei Tang^1,2#^, Hongbo Gao^1,2#^, Dalong Ni^3^, QiFeng Wang^2,4^, Bingxin Gu^2,5,6^, Xinhong He^2,7*^, WeijunPeng^1,2*^

^1^Department of Radiology, Fudan University Shanghai Cancer Center, Shanghai 200032, China.

^2^Department of Oncology, Shanghai Medical College, Fudan University, Shanghai 200032, China.

^3^Departments of Radiology and Medical Physics, University of Wisconsin, Madison, WI, 53705, USA

^4^Department of Pathology, Fudan University Shanghai Cancer Center, Shanghai 200032, China.

^5^Department of Nuclear Medicine, Fudan University Shanghai Cancer Center, Shanghai, 200032,China.

^6^Shanghai Engineering Research Center of Molecular Imaging Probes, Shanghai, 200032,China.

^7^ Department of Interventional Radiology, Fudan University Shanghai Cancer Center, Shanghai 200032, China.

^#^Wei Tang and Hongbo Gao contributed equally to this work.

**Additional Figures**


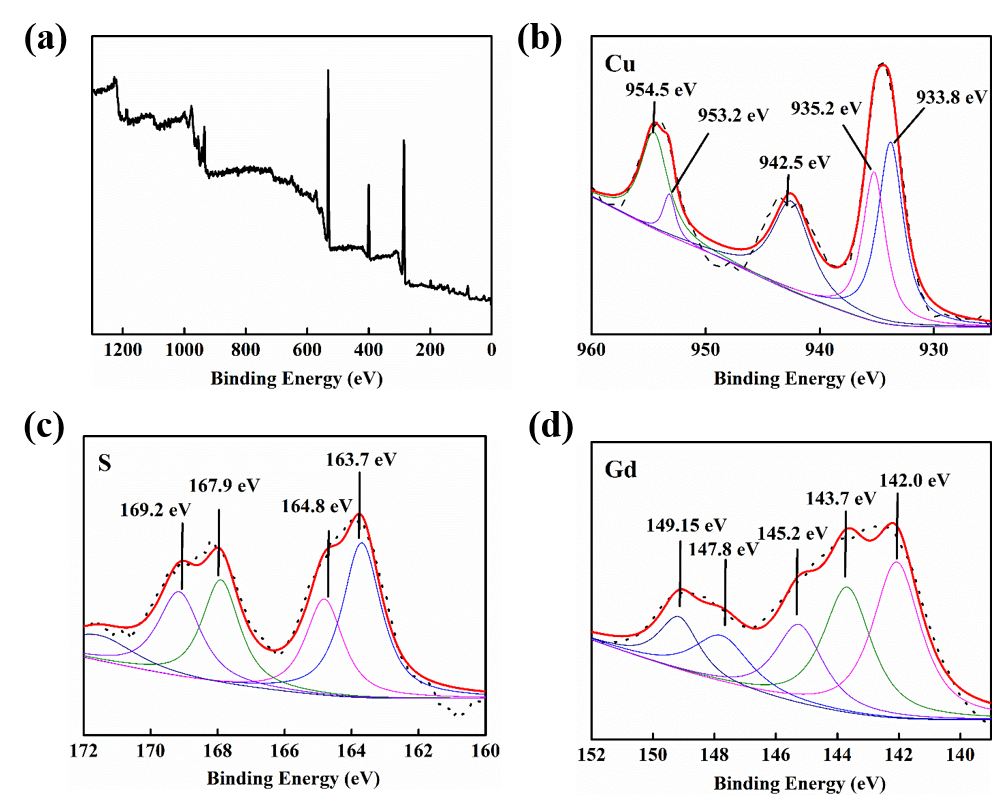


**Additional Figure S1.** X-ray photoelectron spectroscopy (XPS) spectra of a) the nanoparticles, b) Cu 2p, c) S 2p, and d) Gd 4d.


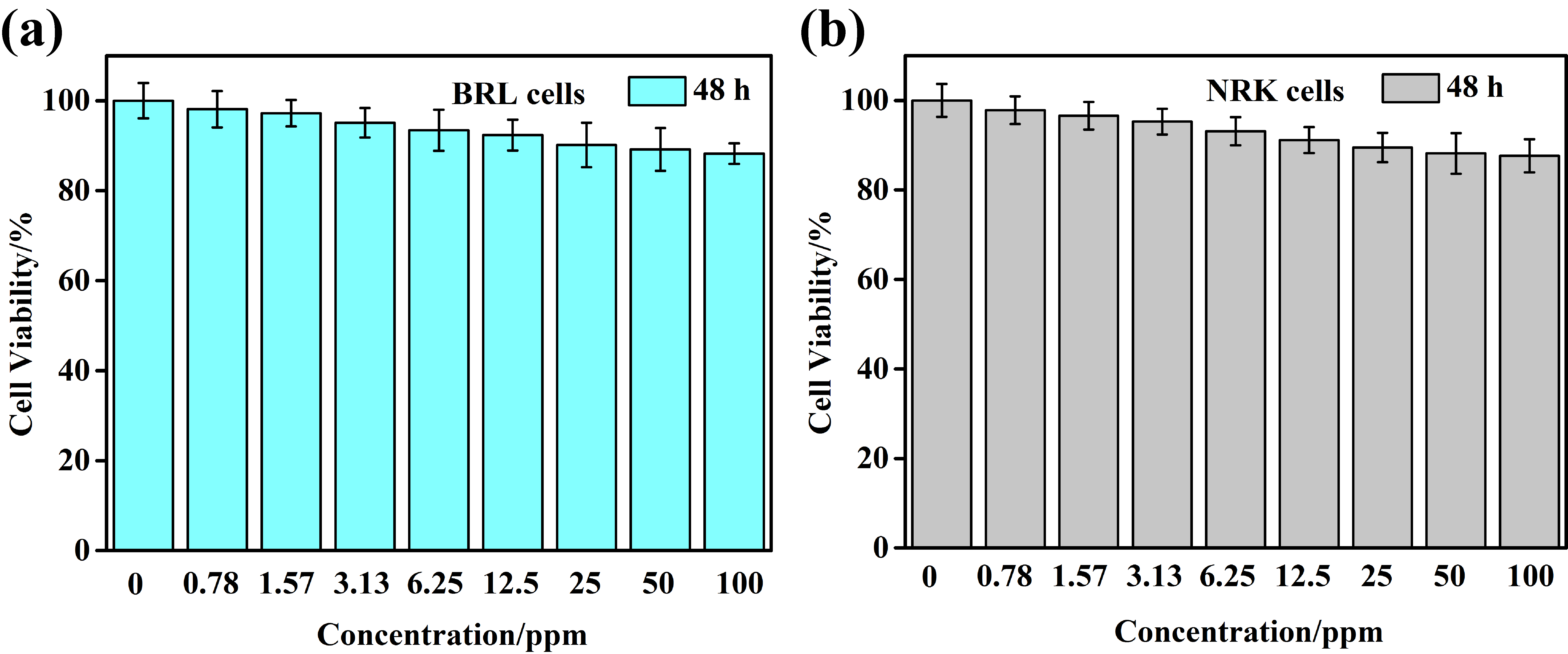


**Figure S2.** Cell viabilities of CuS:Gd NPs with different concentrations on the normal cells (NRK cells and BRL cells) 48 h after co-incubation, further demonstrating the well biocompatibility of CuS:Gd NPs. (n= 6, mean ± s.d.).


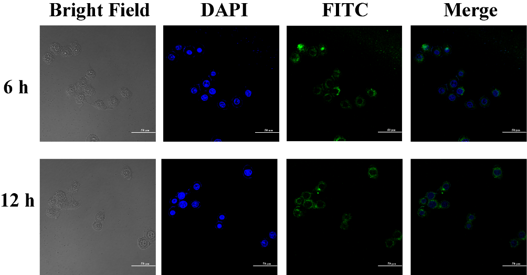


**Additional Figure S3.** Confocal images of 4T1 cells co-incubated with FITC-labeled CuS:Gd NPs (green) for different time points, with the cell nuclei stained by DAPI (blue).


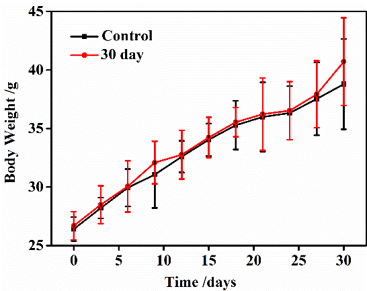


**Additional Figure S4.** Weight measurement of the control group and the CuS:Gd NPs group (30 days after intravenous injection of CuS:Gd NPs)


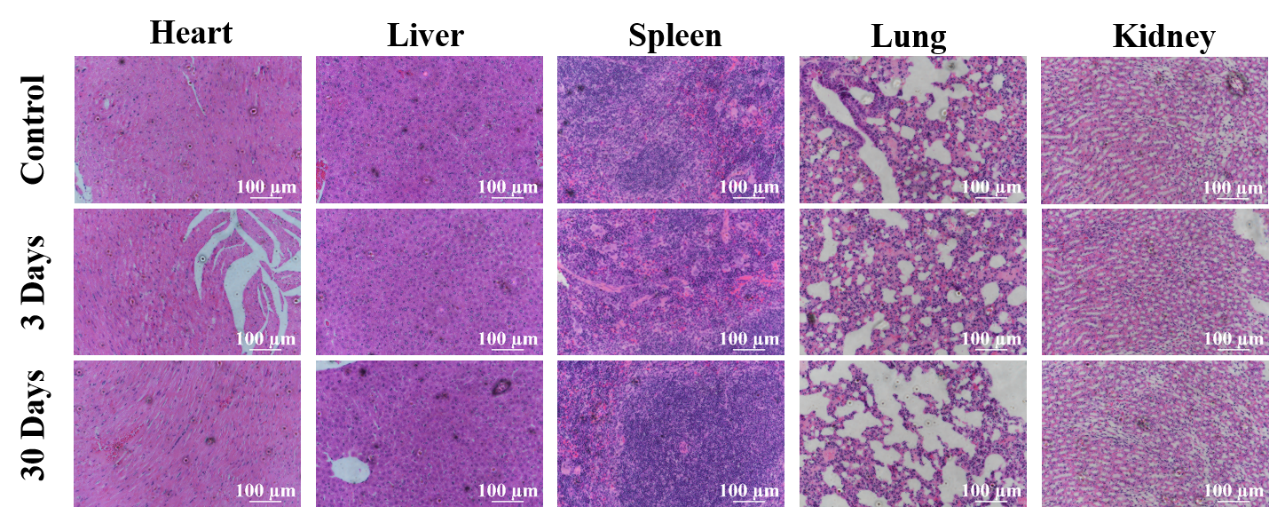


**Additional Figure S5.** Pathological H&E stained images of tissue sections from heart, liver, spleen, lung and kidney of the mice treated with CuS:Gd NPs. The tissue sections were harvested in 3 and 30 days after the intravenous injection of a 25 mg/Kg dosage, showing no significant change of H&E tissue sections.


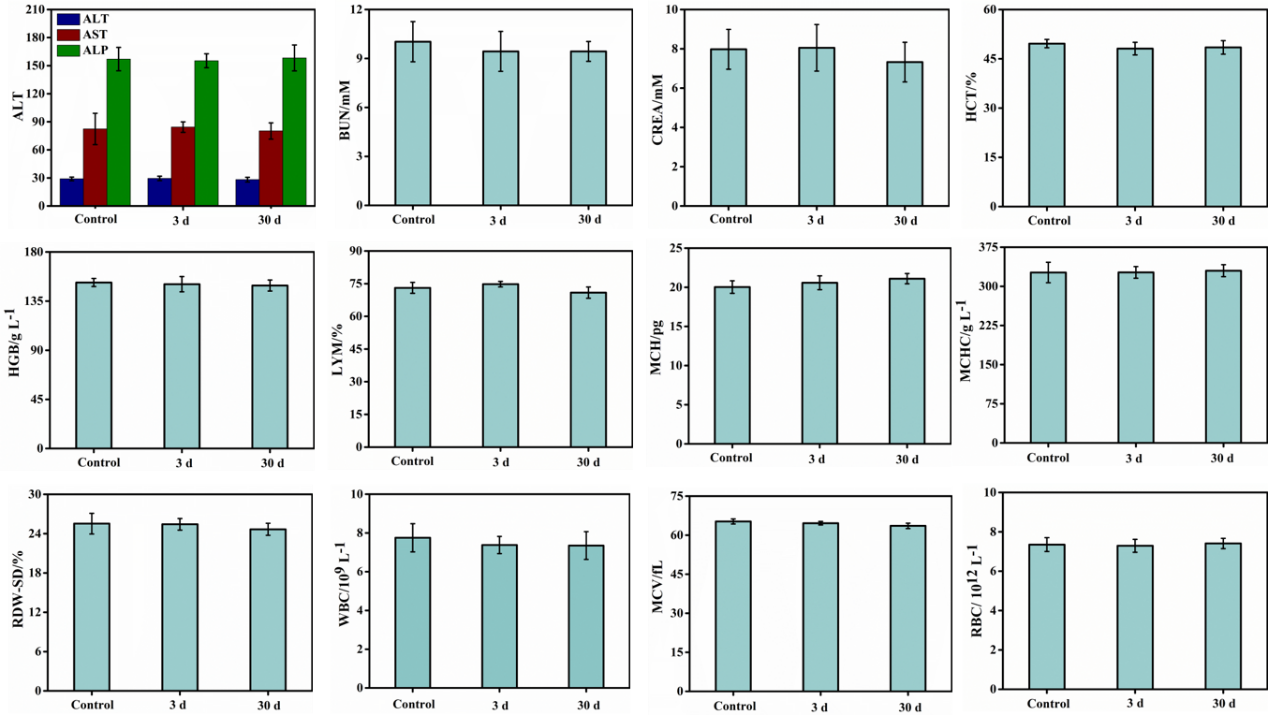


**Additional Figure S6.** Toxicity studies of CuS:Gd NPs *in vivo*. Blood biochemical parameters and hematology data obtained from the mice after the intravenous injection of CuS:Gd NPs (25 mg /Kg , n = 6, mean ± s.d.) in 3 and 30 days with the saline injection as control.


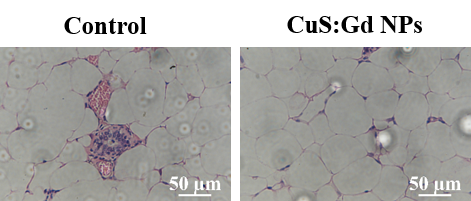


**Additional Figure S7.** H&E staining of the adjacent normal tissues of tumor after the treatment of CuS:Gd NPs, verifying the little toxicity of CDT process on normal cells.
